# Vasoplegia in Cardiac Surgery and Mechanical Circulatory Support: From Cardiopulmonary Bypass to Advanced Circulatory Support Devices

**DOI:** 10.3390/jcdd13080378

**Published:** 2026-08-10

**Authors:** Debora Emanuela Torre, Carmelo Pirri

**Affiliations:** 1 Department of Cardiac Anesthesia and Intensive Care Unit, Cardiac Surgery, Ospedale dell’Angelo, 30174 Venice, Italy; deboraemanuela.torre@aulss3.veneto.it; 2 Department of Neurosciences, Institute of Human Anatomy, University of Padova, 35121 Padua, Italy

**Keywords:** vasoplegia, vasoplegic syndrome, cardiopulmonary bypass, postcardiotomy vasoplegia, vasodilatory shock, mechanical circulatory support, extracorporeal membrane oxygenation, ventricular assist device

## Abstract

Vasoplegia remains one of the most challenging and incompletely understood complications across the spectrum of mechanical circulatory support (MCS). Initially described following cardiopulmonary bypass, it is increasingly recognized in patients supported with veno-arterial extracorporeal membrane oxygenation (V-A ECMO) and combined unloading strategies such as ECPELLA (ECMO and Impella support) as well as in patients bridged to heart transplantation with temporary or durable mechanical circulatory support (MCS). Despite occurring in different clinical settings, these syndromes share common pathophysiological features, including systemic inflammation, endothelial dysfunction, glycocalyx degradation, dysregulated nitric oxide signaling, neurohormonal imbalance, microcirculatory impairment and severe vasomotor dysregulation. Although vasoplegia is commonly considered a technology-specific complication, growing evidence suggests that CPB-associated vasoplegia, postcardiotomy vasoplegic syndrome, ECMO-related vasodilatory shock and distributive shock during ECPELLA may represent distinct manifestations of a common pathobiological process driven by blood–artificial surface interactions, ischemia–reperfusion injury, hemolysis and immune activation. This narrative review proposes a unified framework of vasoplegia across the continuum of MCS. Key mechanistic pathways and current therapeutic strategies, including catecholamines, vasopressin, angiotensin II, methylene blue and hydroxocobalamin, are discussed. By integrating evidence from cardiac surgery, critical care and mechanical circulatory support, vasoplegia is presented as a unifying syndrome of extracorporeal circulation. This perspective may support earlier recognition, phenotype-based management and the development of more targeted therapeutic strategies in a clinically significant yet underexplored area of cardiovascular critical care.

## 1. Introduction

Vasoplegia represents one of the most severe and clinically challenging forms of circulatory dysfunction encountered in contemporary cardiovascular medicine. Characterized by profound systemic vasodilation, markedly reduced systemic vascular resistance, preserved or increased cardiac output and impaired responsiveness to conventional vasopressor therapy, vasoplegia frequently complicates the perioperative and critical care management of patients undergoing cardiac surgery or requiring advanced mechanical circulatory support (MCS) [1,2]. Despite major advances in surgical techniques, extracorporeal technologies and perioperative care, vasoplegic syndromes continue to be associated with substantial morbidity, prolonged intensive care unit and hospital length of stay, increased healthcare resource utilization and excess mortality [3]. Historically, vasoplegia has been most commonly described in the setting of cardiopulmonary bypass (CPB), where exposure of blood to non-endothelial surfaces, surgical trauma, ischemia–reperfusion injury and systemic inflammatory activation contribute to a profound alteration in vascular homeostasis. Consequently, postcardiotomy vasoplegic syndrome has long been regarded as a distinct clinical entity, largely confined to the field of cardiac surgery [3,4]. However, the progressive expansion of temporary and durable MCS technologies over the last two decades has challenged this traditional perspective. Patients supported with veno-arterial extracorporeal membrane oxygenation (V-A ECMO), temporary ventricular assist devices (VADs), extracorporeal cardiopulmonary resuscitation (eCPR) and combined support strategies such as ECPELLA are increasingly recognized to develop hemodynamic profiles remarkably similar to those observed in classical postcardiotomy vasoplegia. In these settings, severe vasodilatory shock may persist despite adequate restoration of systemic blood flow and apparent correction of the primary cardiac insult, frequently requiring escalating doses of vasopressors and adjunctive therapies. Importantly, the occurrence of vasoplegia in MCS-supported patients is not merely a hemodynamic abnormality but rather a complex systemic disorder that may contribute to multiorgan dysfunction, impaired tissue perfusion, renal injury, prolonged support dependency and adverse clinical outcomes [3,5,6,7,8]. The growing adoption of MCS devices has profoundly transformed the epidemiology of cardiovascular critical illness. Patients with refractory cardiogenic shock, postcardiotomy failure, acute myocardial infarction, fulminant myocarditis and advanced heart failure are now routinely managed using increasingly sophisticated extracorporeal support platforms. While these technologies have significantly improved survival in selected populations, they have also introduced novel biological and hemodynamic challenges [5,9]. Among these, vasoplegia has emerged as a recurring and often underrecognized complication that transcends individual device and clinical scenarios. Traditionally, vasoplegia associated with CPB, cardiac surgery, V-A ECMO, VADs and combined unloading strategies has been investigated within separate scientific domains. As a result, the existing literature remains fragmented, with limited integration between cardiac surgery, critical care, extracorporeal life support and mechanical circulatory support research. This compartmentalized approach may underestimate the substantial overlap that exists among these conditions and may hinder the development of unified diagnostic frameworks and therapeutic strategies. Increasing evidence suggests that, despite differences in clinical presentation and support configuration, vasoplegia occurring across the spectrum of extracorporeal circulation and MCS may share a common biological substrate. Rather than representing isolated technology-specific complications, these syndromes may be viewed as different manifestations of a broader pathophysiological continuum characterized by disruption of vascular homeostasis and maladaptive host responses to extracorporeal support [3,6,10]. Such a perspective has important implications, as it may facilitate earlier recognition of high-risk patients, improve mechanistic understanding and promote more individualized therapeutic approaches.

In this narrative review, we provide a comprehensive overview of vasoplegia across the continuum of cardiac surgery and mechanical circulatory support, encompassing cardiopulmonary bypass, postcardiotomy vasoplegic syndrome, V-A ECMO, temporary and durable ventricular assist devices and combined support configurations. We discuss concepts regarding epidemiology, clinical manifestations and management strategies while highlighting the converging mechanisms that may underpin vasoplegia across the seemingly distinct clinical contexts.

## 2. Materials and Methods

This narrative review was designed to synthesize the available evidence on vasoplegia across cardiac surgery and the spectrum of mechanical circulatory support, with particular emphasis on the shared pathophysiological pathways, clinical phenotypes, hemodynamic assessment and therapeutic strategies observed across different extracorporeal support modalities. A structured literature search was conducted in PubMed/MEDLINE, Scopus and Embase from database inception to 20 June 2026. The search strategy included combinations of Medical Subject Headings (MeSH) terms and free-text keywords related to “vasoplegia”, “vasoplegic syndrome”, “postcardiotomy vasoplegia”, “cardiac surgery vasoplegia”, “extracorporeal membrane oxygenation vasoplegia”, and “ventricular assist devices vasoplegia”. Additional relevant studies were identified through manual screening of the reference lists of selected articles and major reviews. Studies were considered eligible when they addressed the epidemiology, definition, pathophysiology, risk factors, diagnostic approach, hemodynamic profile, clinical consequences or treatment of vasoplegia in adult patients undergoing cardiac surgery with cardiopulmonary bypass or receiving temporary or durable mechanical circulatory support, including V-A ECMO and VADs. Original clinical studies, randomized trials, observational studies, mechanistic investigations, systematic reviews, meta-analyses, consensus documents and influential landmark publications were prioritized according to their relevance and methodological contribution. The literature focused primarily on septic or non-cardiovascular vasodilatory shock was considered only when it provided mechanistic or therapeutic insights applicable to vasoplegia occurring during extracorporeal circulation or mechanical circulatory support. Given the heterogeneity of the available literature, the predominance of observational studies and the broad mechanistic and clinical scope of this review, no formal meta-analysis or risk-of-bias assessment was performed. The evidence was therefore synthesized narratively, with particular emphasis on the converging biological pathways and clinical features linking vasoplegia across cardiopulmonary bypass, V-A ECMO, ECPELLA and ventricular assist support.

## 3. Main Sections

### 3.1. Vasoplegia Across the Spectrum of Mechanical Circulatory Support

#### 3.1.1. Definition and Clinical Characteristics of Vasoplegia

Vasoplegia is a distributive form of circulatory shock characterized by profound vasodilation, markedly reduced systemic vascular resistance (SVR), preserved or increased cardiac output and persistent hypotension despite adequate intravascular volume status [1]. Although initially described in the context of septic shock, vasoplegia has become increasingly recognized as a distinct clinical syndrome occurring in patients undergoing cardiac surgery, extracorporeal membrane oxygenation (ECMO) and mechanical circulatory support (MCS). Its hallmark feature is the inability of the vascular system to maintain adequate vasomotor tone despite apparently sufficient cardiac performance and systemic blood flow [2,3,6,8,11,12]. The absence of a universally accepted definition remains one of the major challenges in both clinical practice and research. Various studies have proposed different diagnostic criteria, generally combining persistent hypotension, low SVR and a requirement for vasopressor support in the absence of significant ventricular dysfunction. In the postcardiotomy setting, vasoplegic syndrome is commonly defined by a mean arterial pressure (MAP) below 60–65 mmHg, a cardiac index greater than 2.2 L/min/m^2^, and systemic vascular resistance below 800 dyne s/cm^5^ and is frequently accompanied by significant vasopressor requirements despite adequate preload optimization [1,2,3,13]. However, these thresholds vary considerably across studies, contributing to the wide heterogeneity observed in reported incidence rates [2]. From a hemodynamic perspective, vasoplegia differs substantially from cardiogenic shock. While cardiogenic shock is characterized by inadequate cardiac output and impaired tissue perfusion secondary to ventricular dysfunction, vasoplegia is primarily a disorder of vascular tone [1,14]. Consequently, patients may present with normal or supranormal cardiac output while remaining profoundly hypotensive because of severe reductions in systemic vascular resistance [1,2]. Nevertheless, the distinction between these syndromes is frequently blurred in cardiac surgical and ECMO populations, where myocardial dysfunction and vasodilatory shock often coexist, creating mixed shock states that require comprehensive hemodynamic assessment [3,9,15]. The clinical manifestations of vasoplegia extend beyond systemic hypotension. Persistent vasodilation can impair organ perfusion despite apparently adequate macrocirculatory hemodynamic parameters, reflecting a loss of hemodynamic coherence driven by microcirculatory dysfunction and endothelial injury. Patients frequently require prolonged exposure to high-dose vasopressors, which may further contribute to tissue ischemia, arrhythmias, acute kidney injury and other complications [16,17,18]. Severe vasoplegia has been associated with prolonged mechanical ventilation, increased intensive care unit length of stay, higher healthcare costs and significantly increased mortality [19]. Importantly, although vasoplegia has historically been viewed as a complication of cardiopulmonary bypass, mounting evidence suggests that it represents a broader biological response to extracorporeal circulation and mechanical circulatory support. Similar hemodynamic phenotypes have been described following cardiopulmonary bypass, during ECMO support and after ventricular assist device implantation [6,19,20]. These observations support the concept that vasoplegia may represent a common syndrome arising from shared mechanisms of systemic inflammation, endothelial dysfunction, neurohormonal dysregulation and blood–artificial surface interactions across the continuum of cardiovascular support technologies.

#### 3.1.2. Epidemiology and Clinical Burden of Vasoplegia

**Figure 1:**
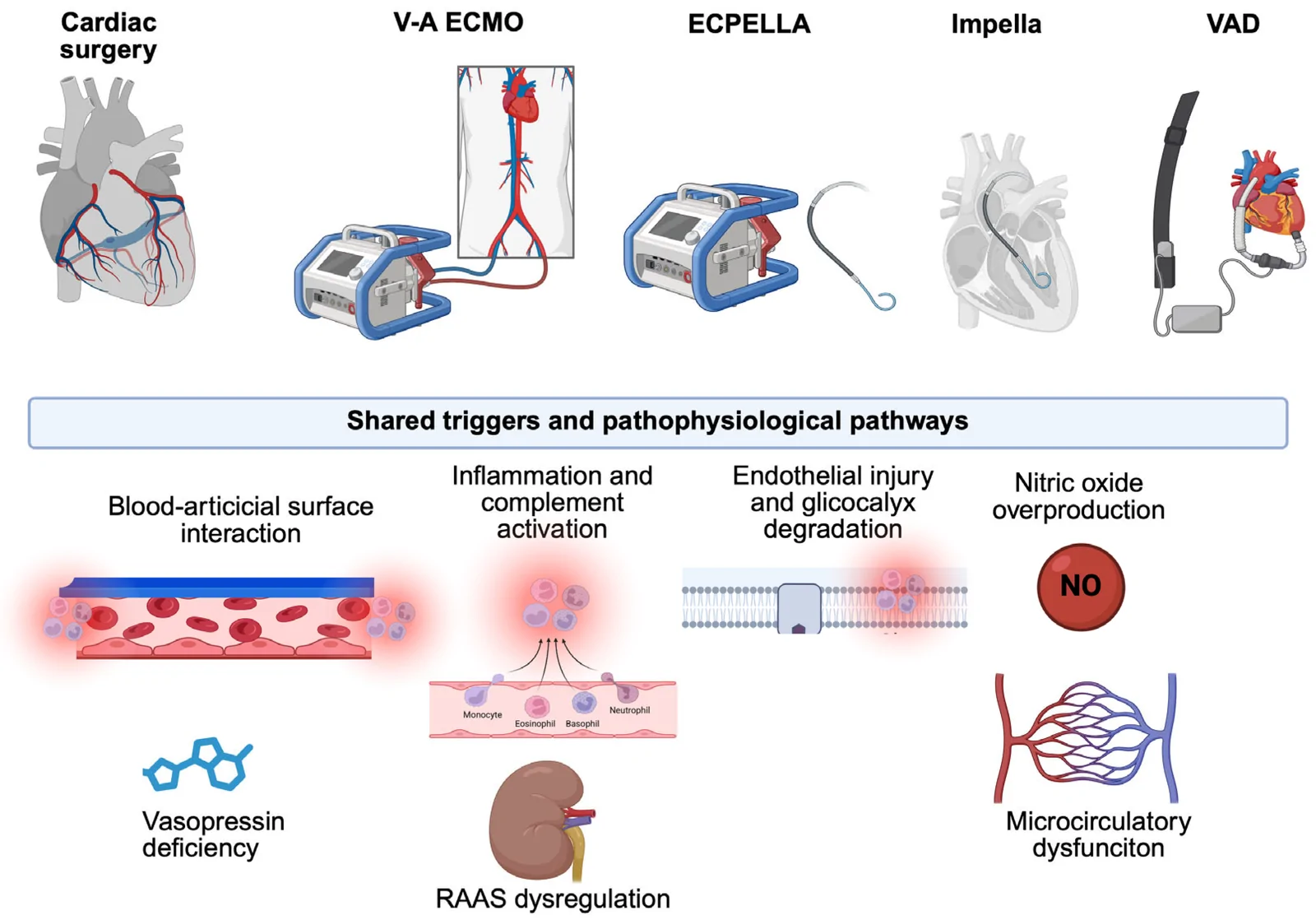
Vasoplegia across the continuum of cardiac surgery and mechanical circulatory support. Created in BioRender. Pirri, C. (2026) https://BioRender.com/204ir85.

The epidemiology of vasoplegia varies considerably according to the clinical setting, patient population and diagnostic criteria employed. In cardiac surgery, vasoplegic syndrome has been reported in approximately 5–25% of patients undergoing procedures with cardiopulmonary bypass, although incidences exceeding 40% have been described in selected high-risk populations, including patients with advanced heart failure, prolonged cardiopulmonary bypass times, complex aortic procedures and preoperative exposure to renin–angiotensin–aldosterone system inhibitors (particularly ACE inhibitors and angiotensin receptor blockers) [3,21]. This marked variability largely reflects the lack of standardized diagnostic criteria and the heterogeneity of study populations. Beyond its frequency, vasoplegia represents a major determinant of adverse postoperative outcomes. Numerous observational studies have demonstrated associations between vasoplegic syndrome and prolonged vasopressor requirements, extended mechanical ventilation, longer intensive care unit and hospital stays, acute kidney injury and increased resource utilization. Importantly, severe vasoplegia requiring high-dose or multiple vasopressor agents has consistently been associated with increased short-and-long-term mortality [3,21,22,23]. The burden of vasoplegia appears even greater among patients requiring advanced mechanical circulatory support (Table 1). In the setting of postcardiotomy shock supported with veno-arterial ECMO, distributive vasodilatory states frequently coexist with myocardial dysfunction, contributing to complex hemodynamic profiles and challenging management decisions. Similarly, systemic vasodilation has been increasingly recognized in patients receiving temporary ventricular assist devices, where inflammatory activation, endothelial injury and neurohormonal disturbances may persist despite restoration of systemic perfusion. Although the true incidence of vasoplegia during ECMO and other forms of mechanical circulatory support remains poorly defined, available evidence suggests that vasodilatory shock is not an uncommon phenomenon and may significantly influence outcomes (Figure 1). In these patients, high vasopressor requirements have been associated with worse clinical outcomes, including impaired end-organ recovery, difficulties in weaning from extracorporeal support and increased mortality [6,24,25,26]. However, the relative contribution of vasoplegia compared with concomitant factors such as infection, persistent systemic inflammation, right ventricular dysfunction and adrenal insufficiency remains incompletely understood.

### 3.2. Pathophysiological Mechanisms of Vasoplegia

**Figure 2:**
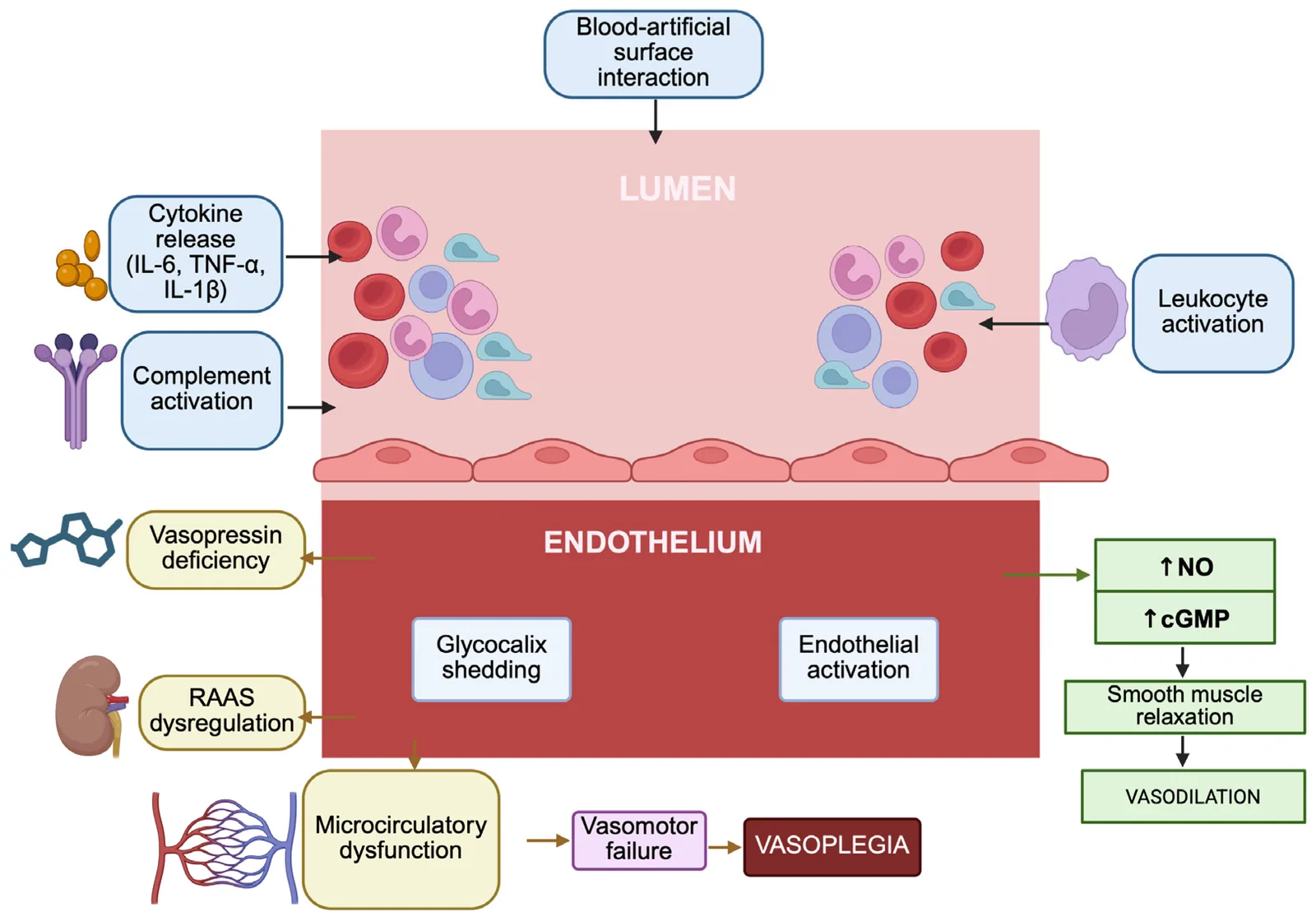
Integrated pathophysiological cascade leading to vasoplegia during cardiopulmonary bypass and mechanical circulatory support. Created in BioRender. Pirri, C. (2026) https://BioRender.com/owpk8bt. Up arrow: increase.

The pathophysiology of vasoplegia is complex, multifactorial and incompletely understood (Table 2). Although historically investigated in the context of cardiac surgery and cardiopulmonary bypass, growing evidence indicates that similar biological mechanisms contribute to vasodilatory shock across the entire spectrum of mechanical circulatory support (Figure 2). Cardiopulmonary bypass, V-A ECMO, VADs and other extracorporeal technologies expose blood to non-physiological surfaces and flow conditions, triggering a systemic response characterized by inflammation, endothelial activation, neurohormonal dysregulation and microcirculatory dysfunction [1,6,27]. Rather than resulting from a single pathogenic pathway, vasoplegia appears to emerge from the interaction of multiple interconnected processes. Blood–artificial surface interactions initiate activation of inflammatory and coagulation cascades, while ischemia–reperfusion injury, surgical trauma, hemolysis and exposure to abnormal shear stress further amplify the host response. These mechanisms ultimately converge on a final common pathway characterized by impaired vascular smooth muscle contractility, excessive vasodilatory signaling, endothelial dysfunction, altered vasopressor responsiveness and disturbances in tissue perfusion [1,3,6,27]. Importantly, the relative contribution of each mechanism may vary according to the underlying clinical scenario, the type and duration of mechanical support and patient-specific factors. Nevertheless, the substantial overlap observed among cardiac surgery, V-A ECMO and VAD populations supports the concept of vasoplegia as a shared pathobiological syndrome rather than a device-specific complication.

#### 3.2.1. Blood–Artificial Surface Interactions

Exposure of circulating blood to artificial biomaterial represents one of the earliest and most important triggers of vasoplegia during extracorporeal circulation and mechanical circulatory support [6,27]. Despite substantial advances in circuit biocompatibility, contact between blood components and non-endothelialized surfaces remains capable of activating multiple inflammatory and coagulation pathways within minutes of support initiation [28]. The adsorption of plasma proteins onto artificial surfaces initiates a complex cascade involving activation of the contact system, complement pathways, platelets, leukocytes and coagulation factors. These events promote the release of numerous pro-inflammatory mediators, including cytokines, chemokines, proteases and reactive oxygen species, generating a systemic inflammatory response that shares many biological and hemodynamic features with sepsis. The magnitude of this response is influenced by factors such as circuit composition, surface coatings, duration of support, blood flow characteristics and patient-specific inflammatory susceptibility [6,27,29]. Cardiopulmonary bypass provides the prototypical example of this phenomenon. However, similar processes occur during prolonged extracorporeal support with V-A ECMO and VADs, where continuous blood exposure to artificial surfaces may sustain inflammatory activation long after implantation. In addition, non-physiological shear stress generated by centrifugal pumps and continuous-flow devices can contribute to cellular activation, endothelial injury and hemolysis, further amplifying the inflammatory milieu [6,27,30,31]. Although blood–artificial surface interactions alone are insufficient to explain the full clinical expression of vasoplegia, they constitute a pivotal upstream event that initiates many of the downstream pathways involved in vasomotor dysfunction. Consequently, they provide a biological foundation upon which complement activation, endothelial injury, nitric oxide dysregulation and neurohormonal alterations subsequently develop.

#### 3.2.2. Complement and Leukocyte Activation

Activation of the complement system represents a central component of the inflammatory response associated with extracorporeal circulation and MCS. Both the classical and alternative complement pathways can be triggered following blood exposure to artificial surfaces, leading to the rapid generation of biologically active mediators that contribute to systemic inflammation and vascular dysfunction. Among these mediators, the anaphylatoxins C3a and C5a play particularly important roles. These molecules promote leukocyte chemotaxis, neutrophil activation, cytokine release, increased vascular permeability and amplification of the inflammatory response [6,32,33,34]. Experimental and clinical studies have consistently demonstrated significant complement activation during cardiopulmonary bypass, with the magnitude of activation correlating with procedural duration and the intensity of the systemic inflammatory response. Leukocyte activation further amplifies this process. Activated neutrophils release proteolytic enzymes, reactive oxygen species and pro-inflammatory cytokines that contribute to tissue injury and vascular dysregulation [29,32,35]. In recent years, increasing attention has been directed toward the role of neutrophil extracellular traps (NETs), which have been implicated in endothelial injury, microvascular dysfunction and thromboinflammatory responses during extracorporeal support. Elevated levels of circulating NET biomarkers have been reported in both cardiac surgical and ECMO populations, suggesting a potential role in the development of organ dysfunction and vasoplegic states [6,36,37,38,39]. Importantly, complement activation is not restricted to the intraoperative period. In patients receiving prolonged V-A ECMO or VAD support, persistent activation of innate immune pathways may contribute to sustained inflammatory signaling, thereby perpetuating vasodilatory responses even after the initial extracorporeal insult has occurred [6,40]. This observation may partly explain why vasoplegia can persist or emerge several days after support initiation despite apparent stabilization of hemodynamic parameters. Overall, complement and leukocyte activation constitute critical early amplifiers of the inflammatory cascade associated with extracorporeal circulation. Their interaction with endothelial cells, vascular smooth muscle and neurohormonal pathways creates the conditions necessary for the subsequent development of vasomotor failure and distributive shock.

#### 3.2.3. Endothelial Dysfunction and Glycocalyx Injury

The vascular endothelium plays a pivotal role in maintaining vascular homeostasis by regulating vasomotor tone, coagulation, permeability, inflammation and microcirculatory function [41]. Increasing evidence suggests that endothelial dysfunction represents a key pathophysiological mechanism underlying vasoplegia across the spectrum of cardiac surgery and MCS [1,3,6]. Exposure to inflammatory mediators, ischemia–reperfusion injury, oxidative stress and abnormal shear forces promotes endothelial activation and disrupts normal vascular regulatory pathways. Activated endothelial cells exhibit increased expression of adhesion molecules, enhanced leukocyte recruitment, altered barrier function and dysregulated production of vasoactive mediators [42,43]. These changes contribute not only to systemic vasodilation but also to capillary leak, tissue edema and impaired organ perfusion. A growing body of evidence has highlighted the importance of glycocalyx degradation in this process. The endothelial glycocalyx is a highly specialized carbohydrate-rich layer that lines the luminal surface of blood vessels and serves as a critical regulator of vascular permeability, mechano-transduction and anti-inflammatory signaling [44,45,46]. During CPB and extracorporeal support, inflammatory activation and oxidative stress induce shedding of glycocalyx components, including syndecan-1 and heparan sulfate, resulting in loss of endothelial integrity and impaired vascular responsiveness. Clinical studies have demonstrated elevated circulating levels of glycocalyx degradation products following cardiac surgery and during ECMO support, with higher concentration frequently associated with greater disease severity and adverse outcomes [47,48,49]. Beyond its role as a biomarker of endothelial injury, glycocalyx disruption may directly contribute to vasoplegia by impairing endothelial–vascular smooth muscle communication and promoting persistent vasodilatory signaling. Importantly, endothelial dysfunction provides a mechanistic bridge linking upstream inflammatory activation to downstream disturbances in vascular tone [50]. Through its effects on nitric oxide bioavailability, vascular reactivity, microcirculatory regulation and neurohormonal signaling, endothelial injury represents a central event in the progression from systemic inflammation to clinically overt vasoplegia.

#### 3.2.4. Nitric Oxide Overproduction and Vasomotor Failure

Among the mechanisms implicated in the development of vasoplegia, dysregulated nitric oxide (NO) signaling is widely regarded as one of the principal determinants of vasomotor failure [1,3,51]. Under physiological conditions, endothelial-derived NO plays a fundamental role in maintaining vascular homeostasis by modulating vascular tone, inhibiting platelet aggregation and regulating leukocyte–endothelial interactions. However, during systemic inflammatory states, excessive NO production can become maladaptive, contributing to profound and persistent vasodilation [51]. Inflammatory cytokines released during CPB, ECMO and MCS stimulate the expression of inducible nitric oxide synthase (iNOS) in endothelial cells, vascular smooth muscle cells and circulating immune cells. Unlike endothelial nitric oxide synthase (eNOS), which produces relatively small amounts of NO under tightly regulated conditions, iNOS generates large and sustained quantities of NO independent of intracellular calcium regulation. The resulting increase in NO bioavailability promotes the excessive activation of soluble guanylate cyclase, leading to the accumulation of cyclic guanosine monophosphate (cGMP) and vascular smooth muscle relaxation. This process contributes directly to the reduction in systemic vascular resistance that characterizes vasoplegic syndrome [3,52,53,54]. Moreover, prolonged exposure to high concentrations of NO may impair vascular responsiveness to endogenous and exogenous vasoconstriction, including catecholamines, thereby contributing to the vasopressor-resistant phenotype frequently observed in severe cases [1,3]. Experimental studies have demonstrated reduced contractile responses of vascular smooth muscles following exposure to inflammation mediators and excessive NO signaling, supporting the concept of acquired vasomotor hyporeactivity [55]. The effects of NO are further amplified by interactions with reactive oxygen species generated during extracorporeal circulation and ischemia–reperfusion injury. The formation of reactive nitrogen species, including peroxynitrite, can exacerbate endothelial dysfunction, impair mitochondrial function and promote cellular injury [56,57]. These mechanisms may contribute not only to systemic vasodilation but also to microcirculatory disturbances and organ dysfunction frequently associated with severe vasoplegia. Evidence supporting the role of NO dysregulation has also influenced therapeutic strategies. The clinical use of methylene blue and, more recently, hydroxocobalamin is largely based on their ability to interfere with NO-mediated signaling pathways and restore vascular tone in selected patients with refractory vasoplegia [1,3,23,58,59,60]. Although the efficacy of these interventions remains an area of ongoing investigation, their clinical effects provide indirect support for the central role of excessive NO activity in the pathogenesis of vasoplegic syndrome.

#### 3.2.5. Relative Vasopressin Deficiency

Arginine vasopressin is a key endogenous regulator of vascular tone and circulatory homeostasis. Under physiological conditions, vasopressin is synthesized in the hypothalamus and released from the posterior pituitary in response to hypotension, hypovolemia and increased plasma osmolality. Through the activation of vascular V1 receptors, vasopressin promotes vasoconstriction independently of adrenergic signaling, thereby contributing to the maintenance of arterial pressure during states of circulatory stress [61]. Accumulating evidence suggests that relative vasopressin deficiency plays an important role in the pathogenesis of vasoplegia [62,63]. Prolonged hemodynamic stress may result in the depletion of pituitary vasopressin stores and an inappropriate decline in circulating hormone levels despite ongoing hypotension. This phenomenon has been described in septic shock and has subsequently been observed in patients undergoing cardiac surgery and extracorporeal circulatory support [62,63,64]. Cardiopulmonary bypass appears to exert significant effects on vasopressin homeostasis. Surgical trauma, systemic inflammatory activation, ischemia–reperfusion injury and neuroendocrine disturbances may collectively impair the physiological vasopressin response. Several clinical studies have reported lower circulating vasopressin concentrations in patients who subsequently develop vasoplegic syndrome compared with those maintaining normal vascular tone, supporting a potential causal relationship between vasopressin depletion and postoperative vasodilatory shock [65]. The importance of this pathway is further supported by the hemodynamic effects of exogenous vasopressin administration. Unlike catecholamines, which rely on adrenergic receptor stimulation, vasopressin restores vascular tone through alternative signaling mechanisms and may remain effective even in the presence of adrenergic receptor desensitization. In addition to its direct vasoconstrictor effects, vasopressin may modulate nitric oxide production, enhance vascular responsiveness to catecholamines and improve arterial pressure with comparatively limited chronotropic activity. These characteristics have contributed to its widespread use as a second-line vasopressor in vasoplegic syndrome following cardiac surgery and during advanced circulatory support [65,66]. Nevertheless, relative vasopressin deficiency should not be viewed as an isolated mechanism. Rather, it represents one component of a broader neurohormonal dysregulation involving multiple vasoconstrictor systems. The interaction between vasopressin depletion, inflammatory activation, endothelial dysfunction and nitric oxide overproduction contributes to the progressive loss of vascular responsiveness that characterizes severe vasoplegia [1,3]. Understanding these interactions is essential for interpreting the heterogeneous clinical response to vasopressor therapy and for developing more targeted hemodynamic strategies. The recognition of vasopressin deficiency as a contributor to vasoplegia has also stimulated renewed interest in the role of other endogenous vasoregulatory pathways, particularly the renin–angiotensin–aldosterone system, which appears to be profoundly altered during CPB, ECMO and MCS.

#### 3.2.6. Renin–Angiotensin–Aldosterone System Dysregulation

The renin–angiotensin–aldosterone system (RAAS) is a fundamental regulator of vascular tone and circulatory homeostasis. Through the generation of angiotensin II, the RAAS contributes to the maintenance of arterial pressure, systemic vascular resistance and organ perfusion during states of hemodynamic stress [67]. Although the role of nitric oxide overproduction and vasopressin deficiency has been extensively investigated in vasoplegic syndrome, growing evidence suggests that alterations in RAAS signaling may also contribute to the development of refractory vasodilation [59,62,68]. Under physiological conditions, reduced renal perfusion and arterial hypotension stimulate renin release, leading to the sequential formation of angiotensin I and angiotensin II. Beyond its potent vasoconstrictor properties, angiotensin II enhances sympathetic activity, promotes aldosterone secretion and supports renal autoregulation. Consequently, impairment of this pathway may compromise the ability of the circulation to respond adequately to systemic hypotension [67]. Several factors associated with cardiac surgery and extracorporeal support may disrupt normal RAAS function. Systemic inflammation, endothelial injury, hemodilution and ischemia–reperfusion injury can alter neurohormonal regulation and vascular responsiveness to endogenous vasoconstrictors. In addition, preoperative treatment with angiotensin-converting enzyme inhibitors (ACE inhibitors) or angiotensin receptor blockers (ARBs), both recognized risk factors for postoperative vasoplegia, may further reduce vasoconstrictor reserve during the perioperative period. Recent studies investigating vasodilatory shock have highlighted the potential importance of relative angiotensin II deficiency [3,68,69]. Elevated plasma renin concentrations have been observed in patients with severe vasodilatory shock and have been associated with worse clinical outcomes, suggesting impaired downstream RAAS activity despite activation of compensatory neurohormonal mechanisms. These findings support the concept that, in some patients, activation of the RAAS may be insufficient to restore vascular tone effectively [70]. Particular interest has emerged in the context of ECMO. Because angiotensin-converting enzyme is predominantly expressed on the pulmonary vascular endothelium, reduced transpulmonary blood flow and pulmonary endothelial dysfunction during V-A ECMO have been proposed as potential mechanisms contributing to impaired conversion of angiotensin I to angiotensin II [71]. Although the clinical significance of this mechanism remains incompletely established, it provides a plausible biological link between extracorporeal support and neurohormonal dysregulation. Beyond its hemodynamic effects, angiotensin II influences endothelial function, microvascular regulation, inflammatory signaling and renal perfusion. Therefore, disturbances of the RAAS may contribute not only to systemic vasodilation but also to the broader pathophysiological consequences of vasoplegia. Collectively, current evidence suggests that RAAS dysregulation represents an important, albeit incompletely understood, component of vasoplegic syndrome and may partly explain the heterogeneity of clinical presentation and response to vasopressor therapy observed across patients undergoing cardiac surgery and MCS [72].

#### 3.2.7. Microcirculatory Dysfunction

While vasoplegia is commonly defined by abnormalities in systemic vascular resistance and arterial pressure, increasing evidence indicates that disturbances at the microcirculatory and cellular levels contribute substantially to its clinical manifestations. Indeed, restoration of macrocirculatory parameters does not necessarily translate into adequate tissue perfusion, suggesting that vascular dysfunction extends beyond the large arterial circulation [16]. Systemic inflammation, endothelial activation, glycocalyx degradation and altered vasoregulatory signaling can disrupt microvascular blood flow, resulting in heterogeneous capillary perfusion and impaired oxygen delivery at the tissue level. Experimental and clinical studies have demonstrated that microcirculatory alterations may persist despite normalization of cardiac output and mean arterial pressure, contributing to ongoing organ dysfunction in critically ill patients [16,73,74]. Similar abnormalities have been described following CPB and during ECMO, where inflammatory activation and endothelial injury are particularly pronounced. In parallel, inflammatory mediators, oxidative stress and reactive nitrogen species may impair mitochondrial function, reducing the ability of cells to utilize delivered oxygen effectively. This phenomenon, often referred to as cytopathic hypoxia, has been implicated in the pathogenesis of multiple organ dysfunction syndromes and may partly explain the discrepancy between apparently adequate systemic perfusion and persistent tissue injury observed in severe vasoplegia [75,76,77]. Although the precise contribution of microcirculatory and mitochondrial dysfunction to vasoplegic syndrome remains incompletely defined, available evidence suggests that these mechanisms represent important downstream consequences of the inflammatory and endothelial disturbances triggered by extracorporeal circulation [77,78]. Their recognition underscores the complexity of vasoplegia as a syndrome involving not only vascular tone abnormalities but also profound alterations in tissue perfusion and cellular energetics.

### 3.3. Vasoplegia After Cardiopulmonary Bypass

#### 3.3.1. Risk Factors

The development of vasoplegia following CPB is influenced by a complex interaction of patient-related, pharmacological and procedural factors. Among pharmacological factors, chronic treatment with angiotensin-converting enzyme inhibitors (ACE inhibitors), angiotensin receptor blockers (ARBs) and, potentially, angiotensin-receptor-neprilysis inhibitors (ARNIs) has consistently been associated with an increased risk of postoperative vasodilatory shock.

In contrast, the contribution of levosimendan appears more complex. Although widely used in selected high-risk cardiac surgical patients because of its inotropic and cardioprotective effects, its intrinsic vasodilatory properties may exacerbate perioperative hypotension and increase vasopressor requirements in susceptible individuals. Nevertheless, current evidence is insufficient to consider levosimendan as an independent risk factor for postoperative vasoplegia [79].

Pre-existing heart failure, reduced left ventricular ejection fraction, chronic kidney disease, advanced age and diabetes mellitus have also been identified as potential risk factors. Several intraoperative variables further contribute to vasoplegia development. Prolonged CPB duration, extended aortic cross-clamp times, complex aortic procedures, redo surgery and extensive transfusion requirements are all associated with greater inflammatory activation and endothelial injury [2,80,81,82].

Beyond serving as a marker of surgical complexity, blood product transfusion may directly contribute to vasoplegia through inflammatory activation, storage lesion-related release of biologically active mediators, nitric oxide pathway dysregulation and endothelial injury. Several studies also suggest a dose-dependent relationship between transfusion burden and postoperative vasoplegia [83,84].

Patients undergoing surgery for infective endocarditis appear to be particularly vulnerable due to the coexistence of systemic inflammation and profound perioperative immune activation [85]. Importantly, no single risk factor is sufficient to predict vasoplegia. Rather, the syndrome appears to result from the cumulative effect of multiple predisposing conditions interacting with the inflammatory and hemodynamic stresses induced by extracorporeal circulation.

#### 3.3.2. Risk Prediction and Risk Stratification

Several predictive models have been developed to identify patients at increased risk of postoperative vasoplegia, with the aim of facilitating perioperative planning and earlier implementation of targeted therapeutic strategies (Table 3). Most models incorporate readily available clinical and procedural variables, although their predictive performances vary considerably across different populations [86,87,88].

Among the available models, the score proposed by van Vessem et al. [86] for patients undergoing surgery for advanced heart failure, including LVAD implantation, demonstrated fair discrimination (AUC 0.759) using variables such as age, anemia, renal function, beta-blocker therapy and type of procedure. More recently, Miles et al. [87] developed a risk score for adult cardiac surgery that stratified patients into low-, intermediate- and high-risk categories according to their probability of developing vasoplegia. However, substantial heterogeneity in vasoplegia definitions, study populations and included variables has limited external validation and widespread clinical adoption. Therefore, current prediction models should be considered complementary tools rather than definitive decision-making instruments and should always be integrated with individualized clinical assessment.

#### 3.3.3. Clinical Consequences

Although often considered a transient postoperative complication, vasoplegia may have substantial clinical consequences. Persistent hypotension and prolonged vasopressor exposure can contribute to impaired tissue perfusion, acute kidney injury, respiratory dysfunction and delayed recovery following cardiac surgery [3,21,22]. Several observational studies have demonstrated an association between severe vasoplegia and longer durations of mechanical ventilation, increased intensive care unit and hospital length of stay and higher healthcare costs. In addition, patients requiring escalating vasopressor support are more likely to develop multiorgan dysfunction and postoperative complications [3,21,22,70]. The prognostic significance of vasoplegia is particularly evident in its most severe forms. Refractory vasoplegic syndrome requiring high-dose or multiple vasopressors has consistently been associated with increased perioperative mortality. Furthermore, persistent vasodilation may complicate the assessment of postoperative hemodynamics by masking concomitant ventricular dysfunction and contributing to mixed shock states that require complex therapeutic management [1]. Given its frequency and clinical impact, early recognition of high-risk patients and prompt implementation of targeted therapeutic strategies remain fundamental components of modern perioperative cardiovascular care.

### 3.4. Vasoplegia During V-A ECMO

Vasoplegia is increasingly recognized as a clinically relevant complication during V-A ECMO. Although ECMO is primarily employed to restore systemic perfusion in patients with refractory cardiogenic shock or postcardiotomy failure, a significant proportion of patients develop a vasodilatory phenotype characterized by low systemic vascular resistance and persistent vasopressor requirements despite adequate extracorporeal blood flow. The pathogenesis of ECMO-associated vasoplegia is multifactorial. In addition to the inflammatory and endothelial disturbances initiated by blood–artificial surface interactions, prolonged extracorporeal support may sustain immune activation through continuous exposure to non-physiological surfaces, hemolysis, altered shear stress and ischemia–reperfusion injury [6,89]. Furthermore, many ECMO patients present with systemic inflammatory states before support initiation, including postcardiotomy shock, myocardial infarction, myocarditis or cardiac arrest, further amplifying the risk of vasodilatory shock. From a clinical perspective, vasoplegia may complicate the interpretation of hemodynamic status during ECMO support. Persistent hypotension despite apparently adequate extracorporeal flow may be mistakenly attributed solely to inadequate circulatory support, potentially leading to unnecessary flow escalation. In contrast, recognition of an underlying vasoplegic component may facilitate more targeted vasopressor and adjunctive therapies. Importantly, severe vasoplegia has been associated with increased vasopressor exposure, impaired end-organ recovery, difficulties in weaning from ECMO and worse clinical outcomes [6,24,89].

### 3.5. Vasoplegia After Heart Transplantation and Bridge to Transplant Mechanical Circulatory Support

Heart transplantation represents a unique clinical setting in which vasoplegia may occur despite satisfactory graft function and in the absence of primary graft dysfunction (PGD). Reported incidences range from approximately 15% to 20%, although estimates vary according to the diagnostic criteria adopted and the characteristics of the transplant population. The coexistence of systemic vasodilation with early graft dysfunction may complicate the hemodynamic assessment, making the distinction between isolated vasoplegia and mixed vasoplegic-cardiogenic shock essential to guide appropriate therapeutic strategies [90,91,92].

The pathophysiology of post-transplant vasoplegia is multifactorial and reflects the cumulative effects of advanced heart failure, prolonged cardiopulmonary bypass, ischemia–reperfusion injury, systemic inflammatory activation and preoperative neurohormonal dysregulation. Additional risk factors include preoperative renal or hepatic dysfunction, prolonged vasopressor support, extensive blood transfusion, previous cardiac surgery and chronic exposure to renin–angiotensin system inhibitors. The contribution of long-term beta-blocker therapy remains controversial, with conflicting evidence reported across observational studies and prediction models [86,87,90,91,93].

Patients bridged to transplantation with temporary or durable mechanical circulatory support represent a particularly high-risk population for postoperative vasoplegia. Durable left ventricular assist device (LVAD) support is associated with chronic endothelial dysfunction, persistent neurohormonal activation, repeated transfusions and increased surgical complexity related to device explantation. In contrast, patients bridged with temporary MCS frequently undergo transplantation in the setting of ongoing cardiogenic shock, systemic inflammation and high vasopressor requirements, all of which may further predispose them to vasodilatory shock after transplantation. Collectively, these factors amplify the perioperative inflammatory burden, exacerbate endothelial dysfunction and increase susceptibility to postoperative vasoplegia [8,88,90,91,94,95].

The differential diagnosis between vasoplegia and PGD is particularly important in the immediate postoperative period. While both conditions may present with hypotension and high vasopressor requirements, isolated vasoplegia is characterized by markedly reduced systemic vascular resistance despite preserved or only mildly impaired graft function, whereas significant ventricular dysfunction suggests the presence of PGD or a mixed shock phenotype. Comprehensive echocardiographic assessment and invasive hemodynamic monitoring are therefore essential to identify the predominant mechanism of circulatory failure. Management generally follows the same principles described for postcardiotomy vasoplegia, including early optimization of preload, prompt initiation of norepinephrine and vasopressin and consideration of rescue therapies such as methylene blue, hydroxocobalamin or angiotensin II in refractory cases. However, treatment should always be individualized, as excessive vasoconstriction or unnecessary escalation of inotropic support may adversely affect graft performance when vasoplegia coexists with early ventricular dysfunction [95].

### 3.6. Vasoplegia in Temporary and Durable Ventricular Assist Device

Vasodilatory syndromes have also been reported in patients receiving temporary and durable ventricular assist devices (VADs), although they remain less extensively studied than postcardiotomy vasoplegia or ECMO-associated vasodilatory shock. The mechanisms involved appear to share many similarities with those observed in other forms of mechanical circulatory support, including systemic inflammation, endothelial activation, neurohormonal dysregulation and altered vascular responsiveness [8,19,30,96]. In temporary VAD recipients, vasoplegia frequently develops in the context of acute cardiogenic shock, where inflammatory activation and tissue hypoperfusion often precede device implantation. Conversely, patients receiving durable continuous-flow left ventricular assist devices may experience chronic alterations in vascular biology resulting from prolonged exposure to non-pulsatile flow and persistent neurohormonal activation. Experimental and clinical studies have demonstrated changes in endothelial function, vascular reactivity and inflammatory signaling following long-term mechanical support, suggesting potential mechanisms through which vasodilatory states may develop [97].

Continuous-flow LVADs expose the vascular system to markedly reduced pulsatility, profoundly altering endothelial mechano-transduction and vascular homeostasis. Physiological pulsatile flow is a major stimulus for endothelial nitric oxide synthase (eNOS) activity, glycocalyx integrity and coordinated microvascular autoregulation. Conversely, chronic exposure to non-pulsatile flow has been associated with impaired endothelial function, glycocalyx degradation, altered nitric oxide signaling, increased oxidative stress and reduced vascular reactivity [98,99,100].

These alterations may contribute not only to chronic endothelial dysfunction but also to an increased susceptibility to vasodilatory states when an additional inflammatory insult, such as surgery or CPB, occurs. Experimental evidence further suggests that restoration of pulsatility improves endothelial function, microvascular perfusion and vascular responsiveness. Consequently, next-generation MCS systems incorporating artificial pulsatility or variable-speed algorithms have been proposed to better reproduce physiological flow patterns and potentially attenuate long-term vascular complications, although their impact on vasoplegia remains to be established [101,102,103,104,105].

Patients undergoing durable LVAD implantation represent one of the highest-risk populations for postoperative vasoplegia. In addition to conventional cardiac surgical risk factors, several device-specific predictors have been identified. A more advanced INTERMACS profile, impaired renal function or preoperative dialysis, prolonged cardiopulmonary bypass duration, repeat sternotomy and greater perioperative transfusion requirements have all been associated with an increased likelihood of postoperative vasoplegia. Preoperative right ventricular dysfunction may further reduce hemodynamic reserve and worsen tolerance to systemic vasodilation, although its role as an independent predictor of postoperative vasoplegia remains uncertain [8,88,94,95].

Collectively, these factors reflect advanced severity of heart failure, chronic endothelial dysfunction, persistent neurohormonal activation and increased surgical complexity that characterize the durable LVAD population.

Although the incidence and prognostic significance of vasoplegia in durable VAD populations remain incompletely defined, persistent vasodilatory shock following implantation has been associated with increased vasopressor requirements, right ventricular dysfunction, renal impairment and prolonged intensive care unit stay [8,106,107,108,109,110]. As the use of temporary and durable MCS continues to expand, improved understanding of device-specific and shared mechanisms of vasoplegia will be essential to optimize hemodynamics and improve clinical outcomes.

### 3.7. Hemodynamic Diagnosis and Differential Diagnosis

The diagnosis of vasoplegia remains primarily clinical and hemodynamic. In its classical presentation, patients exhibit persistent hypotension despite adequate preload, preserved or increased cardiac output, reduced systemic vascular resistance and a requirement for escalating vasopressor support. However, diagnosis is often challenging in critically ill patients, where multiple forms of shock may coexist (Table 4). Particular attention should be paid to distinguish vasoplegia from cardiogenic shock, as the therapeutic implications differ substantially [1,3]. While cardiogenic shock is characterized by inadequate cardiac output resulting from ventricular dysfunction, vasoplegia is primarily a disorder of vascular tone. In practice, mixed shock states are common following cardiac surgery and during mechanical circulatory support, necessitating comprehensive hemodynamic evaluation. Pulmonary artery catheterization remains the most complete tool for differentiating vasoplegia from low-output states, allowing direct assessment of cardiac output, filling pressures and systemic vascular resistance. Echocardiography is equally important and provides rapid bedside evaluation of ventricular function, preload status, valvular abnormalities and potential mechanical complications. Additional markers of tissue perfusion, including serum lactate, venous oxygen saturation, capillary refill time and urine output, may assist in determining the severity of circulatory dysfunction. Another important diagnostic challenge is the differentiation between vasoplegia and sepsis [111,112,113]. Both conditions may present with hypotension, vasodilation, elevated inflammatory markers and vasopressor dependency. In postoperative cardiac surgical patients and individuals receiving mechanical circulatory support, the distinction may be particularly difficult because systemic inflammatory activation frequently occurs in the absence of infection. Careful microbiological assessment and serial clinical evaluation are therefore essential before attributing vasodilatory shock solely to infectious causes [114]. Given the absence of universally accepted diagnostic criteria, early recognition relies on integrating clinical findings, hemodynamic monitoring and assessment of end-organ perfusion. Such an approach is fundamental for the timely initiation of appropriate therapy and the avoidance of mechanical support.

### 3.8. Therapeutic Strategies

The management of vasoplegia remains largely supportive and is primarily aimed at restoring adequate tissue perfusion while minimizing the adverse consequences of prolonged vasopressor exposure (Table 5). Given the heterogeneity of clinical presentations and the frequent coexistence of multiple shock phenotypes, treatment should be guided by comprehensive hemodynamic assessment rather than arterial pressure alone [1].

#### 3.8.1. General Principles of Hemodynamic Management

The initial management of vasoplegia should focus on optimization of effective circulating volume and exclusion of reversible causes of hypotension [1]. Excessive fluid administration should be avoided, particularly in patients undergoing cardiac surgery or receiving MCS, where fluid overload may worsen pulmonary edema, right ventricular dysfunction and organ congestion. Current evidence favors a restrictive, goal-directed approach based on dynamic hemodynamic assessment. Balanced crystalloids remain the preferred first-line resuscitation fluids, whereas the routine use of synthetic colloids is discouraged because of their association with renal injury and coagulation abnormalities. Albumin may be considered in selected patients with severe hypoalbuminemia or marked capillary leak, although robust evidence supporting its routine use specifically in vasoplegia is lacking [115,116,117].

Hemodynamic targets should prioritize adequate end-organ perfusion rather than achievement of arbitrary blood pressure values [118,119]. Furthermore, restoration of arterial pressure should not be pursued at the expense of excessive vasopressor exposure. High cumulative vasopressor doses may contribute to myocardial oxygen consumption, arrhythmias, digital and mesenteric ischemia, microcirculatory dysfunction and organ injury [120]. Consequently, contemporary management strategies increasingly aim to achieve adequate tissue perfusion while minimizing overall vasopressor burden through the early use of adjunctive non-catecholaminergic therapies [3,66]. Mean arterial pressure goals should therefore be individualized according to pre-existing hypertension, ventricular function and evidence of tissue hypoperfusion. Serial assessment of lactate clearance, urine output, capillary refill time and end-organ function may provide valuable information regarding the adequacy of resuscitation [16,17,113]. Importantly, persistent hypotension should not automatically be interpreted as evidence of insufficient cardiac support. Particularly in patients receiving V-A ECMO or other forms of MCS, persistent hypotension is frequently multifactorial and should not automatically be attributed to inadequate circulatory support. Vasoplegia may coexist with residual cardiogenic shock, right ventricular dysfunction, hypovolemia or device-related hemodynamic abnormalities, generating mixed shock states. Accurate differentiation among these conditions is essential because management strategies differ substantially [1,6,121]. While cardiogenic shock may require optimization of ventricular unloading, inotropic support or escalation of mechanical circulatory support, isolated vasoplegia primarily requires restoration of vascular tone through vasopressor and adjunctive therapies. Similarly, hypovolemia necessitates volume resuscitation, whereas right ventricular dysfunction may require targeted interventions aimed at reducing right ventricular afterload and improving contractility [110,122,123]. Failure to identify the predominant mechanism of circulatory failure may lead to inappropriate therapeutic escalation and delayed hemodynamic stabilization.

#### 3.8.2. Vasopressor Therapy

Norepinephrine remains the first-line vasopressor for the treatment of vasoplegia and is recommended by most contemporary critical care and cardiac surgical practices. Its predominant alpha-adrenergic activity effectively increases systemic vascular resistance while minimizing excessive chronotropic stimulation [11]. Nevertheless, some patients develop progressive vasopressor requirements despite escalating norepinephrine doses, a phenomenon frequently observed in severe postcardiotomy vasoplegia and during prolonged extracorporeal support. In such cases, vasopressin is commonly introduced as a second-line agent [124]. Because its mechanism of action is independent of adrenergic receptors, vasopressin may restore vascular tone while reducing catecholamine exposure. This catecholamine-sparing effect is particularly attractive in patients at risk of arrhythmias, myocardial ischemia or right ventricular dysfunction [125,126]. Angiotensin II has recently emerged as a promising option for selected patients with refractory vasodilatory shock. Although clinical experience in cardiac surgery and MCS remains limited compared with septic shock populations, available reports suggest that angiotensin II may effectively increase arterial pressure and reduce vasopressor requirements in carefully selected patients [70,71,127,128]. Further studies are required to better define its optimal timing and role within contemporary treatment algorithms. Emerging evidence suggests that early recognition of vasoplegia and timely introduction of adjunctive non-catecholaminergic therapies may facilitate hemodynamic stabilization and reduce prolonged exposure to high-dose catecholamines, although the optimal timing and sequencing of multimodal vasopressor strategies remain incompletely defined [66,129]. Regardless of the specific agent employed, progressive vasopressor escalation should prompt reassessment of the underlying hemodynamic profile and consideration of adjunctive therapies rather than unlimited dose escalation.

#### 3.8.3. Rescue Therapies and Vasoplegia During MCS

Methylene blue remains the most widely used rescue therapy for refractory vasoplegia following cardiac surgery. Several observational studies and small randomized trials have demonstrated improvements in arterial pressure and reduction in vasopressor requirements following administration, particularly when treatment is initiated early in the course of vasodilatory shock. However, variability in patient selection and study design continues to limit definitive conclusions regarding its impact on survival [20,58,59,130].

Hydroxocobalamin has gained increasing attention as an alternative rescue therapy. Although evidence remains limited to observational experiences and case series, favorable hemodynamic responses have been reported in patients with severe vasoplegia refractory to conventional vasopressors and methylene blue. Whether hydroxocobalamin should be considered before or after methylene blue remains uncertain [59,60,131].

The role of corticosteroids is less clearly defined. While they may improve vascular responsiveness in selected patients, routine administration cannot currently be recommended on the basis of available evidence. Their use is generally reserved for persistent vasopressor dependency or suspected adrenal dysfunction [132].

Management becomes particularly challenging in patients receiving V-A ECMO, ECPELLA or VADs. In these settings, persistent hypotension is often multifactorial, and increasing extracorporeal blood flow alone rarely corrects isolated vasoplegia [6,133,134]. Instead, treatment requires integration of invasive hemodynamic monitoring, echocardiographic assessment and evaluation of tissue perfusion to identify the relative contribution of vasodilatory shock, ventricular dysfunction and ongoing inflammatory processes [75]. Recognition of vasoplegia as a distinct component of shock is especially important during weaning from MCS, where uncontrolled vasodilation may complicate assessment of native cardiac recovery and contribute to weaning failure. In patients undergoing V-A ECMO weaning trials, persistent vasoplegia may compromise the reduction in extracorporeal blood flow despite adequate recovery of native cardiac function [24]. Consequently, restoration of vascular tone should be considered an integral component of the weaning process rather than merely a treatment of hypotension. Failure to recognize an ongoing vasoplegic state may lead to underestimation of myocardial recovery and unnecessary prolongation of mechanical support [135,136]. Given the multifactorial nature of vasoplegia, no single pharmacological intervention is likely to be universally effective. Rather, treatment should be individualized according to the predominant underlying mechanisms, including inflammatory activation, nitric oxide overproduction, neurohormonal dysregulation and concomitant ventricular dysfunction. Notably, most therapeutic strategies currently adopted for vasoplegia during V-A ECMO and other forms of MCS have been extrapolated from the postcardiotomy and critical care literature. Dedicated studies specifically addressing vasoplegia in the MCS population remain scarce, highlighting an important area for future investigation.

## 4. Discussion

Traditionally, vasoplegia has been investigated as a complication specific to individual clinical settings, most notably CPB and cardiac surgery. However, the growing body of evidence reviewed in this manuscript suggests that this perspective may be overly restrictive [1,3,6,24]. Despite differences in patient populations, support configurations and underlying cardiovascular diseases, vasoplegia occurring after CPB, during V-A ECMO and in patients receiving VADs shares many common biological and hemodynamic characteristics [3,6,24,96,97]. These observations support the concept that vasoplegia should be viewed not merely as a technology-specific complication but rather as a shared pathobiological syndrome that may emerge throughout the continuum of MCS [1,6,24]. This conceptual shift has important clinical implications. First, it encourages clinicians to recognize vasoplegia as a distinct contributor to circulatory failure rather than attributing persistent hypotension exclusively to inadequate cardiac function or insufficient extracorporeal support [14,24,135]. This distinction is particularly relevant in patients receiving V-A ECMO or combined support strategies, where vasodilatory shock frequently coexists with varying degrees of myocardial dysfunction [14,15]. Failure to identify the vasoplegic component may lead to the unnecessary escalation of MCS while delaying more appropriate pharmacological interventions [24,136]. Second, the available evidence highlights the persistent lack of standardized diagnostic criteria [1,3,11,121]. Although low systemic vascular resistance, preserved cardiac output and vasopressor dependency are widely recognized hallmarks of vasoplegia, substantial heterogeneity remains across studies. This variability complicates comparisons between investigations, contributes to the wide range of reported incidence rates and may partially explain the limited reproducibility of therapeutic studies [1,3,98]. The development of consensus diagnostic definitions should therefore be considered a research priority.

Another important observation emerging from the current literature is the discrepancy between the increasing clinical relevance of vasoplegia and the relative scarcity of high-quality evidence guiding management [3,12,24]. While norepinephrine, vasopressin, methylene blue and, more recently, hydroxocobalamin and angiotensin II are increasingly used in clinical practice, most available data derive from observational studies, retrospective analyses or small single-center experiences [3,65]. This limitation is particularly evident in the field of MCS, where dedicated studies remain sparse despite the growing use of V-A ECMO, ECPELLA and VADs.

Finally, the pathophysiological complexity of vasoplegia suggests that a “one-size-fits-all” therapeutic approach may be inadequate. The heterogeneous interplay among inflammation, endothelial dysfunction, neurohormonal alterations and microcirculatory disturbances likely generates different biological phenotypes that may respond differently to available therapies [1,70,73,74]. Future management strategies may therefore benefit from a more individualized approach based on patient-specific mechanisms rather than solely on hemodynamic manifestations.

Overall, the evidence reviewed herein supports a broader interpretation of vasoplegia as a syndrome that transcends individual devices and clinical settings. Recognizing the shared biological pathways linking cardiac surgery, extracorporeal circulation and advanced MCS may facilitate earlier diagnosis, improve therapeutic decision-making and stimulate the development of more targeted interventions.

## 5. Future Directions

Despite suboptimal advances in the understanding of vasoplegic syndrome, important knowledge gaps remain across the spectrum of cardiac surgery and MCS. One of the major challenges is the absence of universally accepted diagnostic criteria, which continues to limit comparisons among studies and hinders the development of standardized therapeutic algorithms. Future efforts should focus on establishing consensus definitions capable of integrating hemodynamic, biochemical and clinical parameters. Growing interest has also emerged in the identification of biomarkers that may facilitate earlier diagnosis, risk stratification and therapeutic guidance. Markers of endothelial injury and glycocalyx degradation, including syndecan-1, angiopoietin-2 and endocan, have shown promising associations with disease severity. Similarly, biomarkers reflecting neurohormonal dysregulation, such as plasma renin and angiotensin II levels, may contribute to the identification of distinct biological phenotypes of vasoplegia. However, their clinical utility remains to be validated in prospective studies. Another important area of investigation concerns the application of precision medicine approaches. Vasoplegia is increasingly recognized as a heterogeneous syndrome resulting from the interaction of multiple pathophysiological pathways rather than a single disease entity. Future research should aim to identify specific endotypes characterized by predominant inflammatory, endothelial, neurohormonal or microcirculatory disturbances, potentially enabling more individualized therapeutic strategies. Finally, dedicated studies are urgently needed in patients receiving advanced MCS.

Finally, dedicated studies are urgently needed in patients receiving advanced MCS. Further research is also warranted to better characterize vasoplegia in patients bridged to heart transplantation with temporary or durable MCS, with particular emphasis on risk stratification, pathophysiological mechanisms and individualized therapeutic approaches.

Most current therapeutic approaches have been extrapolated from the postcardiotomy and critical care literature, while high-quality evidence in V-A ECMO, ECPELLA and VAD populations remains scarce. As the use of these technologies continues to expand, improved understanding of device-specific and shared mechanisms of vasoplegia will be essential to optimize patient selection, hemodynamic management and clinical outcomes.

## 6. Conclusions

Vasoplegia should no longer be regarded solely as a complication of cardiopulmonary bypass. Rather, it represents a shared syndrome of circulatory failure that may develop throughout the continuum of MCS. Recognizing the common biological pathways linking cardiac surgery, V-A ECMO and VADs may improve diagnostic accuracy, guide therapeutic decision-making and support the development of more personalized treatment strategies in cardiovascular critical care.

## Figures and Tables

**Table 1:** Clinical characteristics of vasoplegia across different mechanical circulatory support settings. CPB: cardiopulmonary bypass; CO: cardiac output; SVR: systemic vascular resistance; AKI: acute kidney injury; V-A ECMO: veno-arterial extracorporeal membrane oxygenation; ECPELLA: eCMO and IMPELLA support; VAD: ventricular assist devices; RV: right ventricle; ICU: intensive care unit.

Setting	Typical Timing	Predominant Hemodynamic Profile	Main Clinical Consequences
CPB	Immediate postoperative period	Low SVR with preserved/high CO	AKI, prolonged ventilation
V-A ECMO	During support	Low SVR despite adequate ECMO flow; mixed shock may coexist	Difficult weaning, organ dysfunction
ECPELLA	During combined support	Low SVR despite combined support and LV unloading; mixed shock can coexist	Complex hemodynamic management
Temporary VAD	Early post-implant	Low SVR with inflammatory activation despite mechanical support; mixed shock may coexist	Vasopressor dependency
Durable LVAD	Early and late phases	Chronic vascular dysregulation associated with continuous flow support	RV dysfunction, prolonged ICU stay
Heart transplantation	Early after graft reperfusion and separation from CPB	Low SVR despite preserved graft function; mixed vasoplegic-graft dysfunction phenotype may coexist	High vasopressor requirements, AKI, prolonged mechanical ventilation, difficult assessment of graft function, prolonged ICU stay

**Table 2:** Pathophysiological mechanisms of vasoplegia. SVR: systemic vascular resistance; RAAS: renin–angiotensin–aldosterone system.

Mechanism	Principal Effect	Clinical Relevance
Blood–artificial surface interaction	Inflammatory activation	Triggering event
Complement activation	Cytokine release	Amplification of inflammation
Endothelial dysfunction	Loss of vascular homeostasis	Vasodilation and capillary leak
Glycocalyx degradation	Increased permeability	Tissue edema
Nitric oxide overproduction	Vasomotor failure	Reduced SVR
Relative vasopressin deficiency	Reduced vasoconstrictor reserve	Vasopressor dependency
RAAS dysregulation	Impaired vascular responsiveness	Refractory vasoplegia
Microcirculatory dysfunction	Altered tissue perfusion	Organ dysfunction

**Table 3:** Published prediction models for vasoplegia after cardiac surgery and durable mechanical circulatory support implantation. LVAD: left ventricular assist device; INTERMACS: interagency registry for mechanically assisted circulatory support; CPB: cardiopulmonary bypass; AUC: area under the receiver operating characteristic curve; MELD: model for end-stage liver disease; LVEF: left ventricular ejection fraction; CABG: coronary artery bypass grafting.

Study	Population	Predictors Included	Main Findings	Major Limitations
Van Vessem et al. [86]	225 patients undergoing advanced heart failure surgery, including durable LVAD implantation	Sex, prior hypertension, NYHA class 3 or 4, pulmonary hypertension, previous cardiac surgery procedure, EuroSCORE II, anemia, creatinine clearance, serum thyroxine, ACE inhibitors/ARB therapy, inotropes Beta-blocker therapy associated with lower risk of vasoplegia	Good discrimination (AUC: 0.759); vasoplegia incidence increased across low- (13%), intermediate- (39%) and high-risk (65%) groups	Single-center derivation cohort; no external validation; advanced heart failure population only
Miles et al. [87]	Adult patients undergoing cardiac surgery	Older age, sex (male), LVEF, previous cardiac surgery, heart failure, preoperative dialysis, liver disease, preoperative amiodarone, preoperative beta-blockers, non-elective surgery, combined CABG-valve surgery, CPB time	Stratified patients into low- (3.9%), intermediate- (14.8%) and high-risk (34.2%) groups for postoperative vasoplegia	Retrospective derivation; heterogeneous surgical population; not validated in MCS or transplantation
Tecson et al. [88]	252 patients undergoing continuous-flow LVAD implantation	INTERMACS profile, systolic blood pressure, central venous pressure, CPB duration	Identified preoperative and intraoperative predictors associated with postoperative vasoplegia	Predictive model rather than a validated bedside risk score; limited external validation
Lamba et al. [8]	600 patients undergoing continuous-flow LVAD implantation	Sex, preoperative stroke, preoperative dialysis, body mass index, higher neutrophil:lymphocyte ratio, higher MELD score	Prolonged vasoplegia occurred in 30.3% of patients and was independently associated with increased 30-day mortality	Retrospective single-center study

**Table 4:** Differential diagnosis of vasoplegia. MAP: mean arterial pressure; SVR: systemic vascular resistance; ↓: decrease.

Variable	Vasoplegia	Cardiogenic Shock	Septic Shock
MAP	↓	↓	↓
Cardiac output	Normal/high	Low	Normal/high
SVR	Low	High/normal	Low
Filling pressures	Variable	Often elevated	Variable
Ventricular function	Preserved or mildly impaired	Severely impaired	Usually preserved
Vasopressor requirement	High	Variable	High
Infection	Absent	Absent	Present

**Table 5:** Pharmacological therapies for vasoplegia: mechanisms of action, clinical roles and major limitations. RAAS: renin–angiotensin–aldosterone system; MCS: mechanical circulatory support; NO: nitric oxide; cGMP: cyclic guanosine monophosphate; G6PD: glucose-6-phosphate dehydrogenase.

Therapy	Mechanism of Action	Clinical Role	Main Limitations
Norepinephrine	Alpha-adrenergic agonist	First-line vasopressor	Tachyarrhythmias, increased myocardial oxygen demand, excessive vasoconstriction at high doses, no correction of the underlying pathophysiology
Vasopressin	V1 receptor agonist	Catecholamine-sparing agent	Digital and mesenteric ischemia, reduced cardiac output in susceptible patients, limited evidence regarding optimal timing and dosing
Angiotensin II	RAAS restoration	Refractory vasoplegia	Limited evidence in cardiac surgery and MCS, potential thromboembolic risk, high cost, uncertain optimal patient selection
Methylene blue	Inhibition of NO-cGMP pathway	Rescue therapy	Risk of serotonin syndrome, contraindicated in G6PD deficiency, interference with pulse oximetry, uncertain optimal timing and dosing
Hydroxocobalamin	Nitric oxide scavenging	Rescue therapy	Limited randomized evidence, interference with laboratory assays, chromaturia, possible oxalate nephropathy
Corticosteroids	Improved vascular responsiveness and modulation of inflammation	Adjunctive therapy	Heterogeneous evidence, hyperglycemia, increased infection risk, uncertain patient selection and optimal treatment duration

## Data Availability

No new data were created or analyzed in this study. Data sharing is not applicable to this article.

## Abbreviations

The following abbreviations are used in this manuscript:

VAD	Ventricular assist device
MCS	Mechanical circulatory support
ECPELLA	ECMO and Impella support
CPB	Cardiopulmonary bypass
LVAD	Left ventricular assist device
ECMO	Extracorporeal membrane oxygenation
